# Microbial pollution and food safety

**DOI:** 10.3934/microbiol.2018.3.377

**Published:** 2018-06-05

**Authors:** Thomas Bintsis

**Affiliations:** Department of Agricultural Technology, TEI of West Macedonia, Florina, Greece

**Keywords:** microbial pollution, environmental contamination, foodborne pathogen outbreaks, food safety, meat production, fresh produce

## Abstract

Microbial pollution is a serious food safety issue because it can lead to a wide range of foodborne diseases. A great number of foodborne diseases and outbreaks are reported in which contamination of fresh produce and animal products occurs from polluted sources with pathogenic bacteria, viruses and protozoa and such outbreaks are reviewed and the sources are revealed. Investigations of foodborne outbreaks involved meat production and fresh produce, namely, that occurred at the early stages of the food chain have shown certain sources of contamination. Domesticated food animals, as well as wild animals, flies and rodents can serve as a source of contamination of nearby produce-growing fields and can lead to human infection through direct contact at farms and, mostly, mail order hatcheries. The most of the fresh produce associated outbreaks have followed wildlife intrusion into growing fields or fecal contamination from nearly animal production facilities that likely led to produce contamination, polluted water used for irrigation and improper manure. Preventive measures, as part of implemented good agricultural practice systems are described. Controlling and minimizing pre-harvest contamination may be one of the key aspects of food safety.

## 1. Introduction

Microbial pollution is an environmental problem and during the past decades the microbial pollution has been increasing and is considered as important issue in food security. Microbial pollution is a serious issue because it can lead to a wide range of health problems [1]. A great number of foodborne diseases and outbreaks are reported in which contamination of fresh produce and animal products occurs from polluted sources with pathogenic bacteria, viruses and protozoa [2]. Besides diseases and death, the consumption of pathogen contaminated foods also creates economic impact that can be quite devastating on the consumers, a nation, food dealers and food companies [2]. Pathogenic bacteria, viruses and protozoa could be introduced to the foods of both animal and non-animal products during: (1) primary production (in the farm where plants are grown or animals are raised for food; (2) at harvest and slaughter of food produce and food animals respectively; (3) transportation; (4) food processing; (5) storage; (6) distribution and (7) preparation and serving (both outside and inside home). Since the most common sources of environmental pollution with microorganisms occur in the primary production, the current review focus on the first stage of the food chain. Therefore, the sources of contamination caused by microbial pollution are reviewed, together with the suggested measurements to control the contaminations at the earlier stages of the food chain.

Outbreak investigations have revealed that contamination from polluted sources occur throughout the long food chain during the production of fresh produce and livestock, that is, while growing the plants or raising food animals [3]. Many factors throughout all stages of the food production and distribution system can affect food safety. However, since the microbial pollution affects the food chain at the early stages, that is the pre-harvest ones, the current review focuses on the animal production before slaughter and the growing of fresh produce.

## 2. Outbreak investigations—animal products

For meat products, what happens on farms, in feedlots, during transport and before slaughter can have a major effect on human health [4]. Domesticated food animals can serve as a source of contamination of nearby produce-growing fields and can lead to human infection through direct contact at petting farms and mail order hatcheries. CDC, multiple states, and the US Department of Agriculture's Animal and Plant Health Inspection Service investigated eight separate multistate outbreaks of human *Salmonella* infections linked to contact with live poultry in backyard flocks [5]. In the eight outbreaks, 895 people infected with the outbreak strains of *Salmonella* were reported from 48 states in 2016. Epidemiologic, traceback, and laboratory findings linked the eight outbreaks to contact with live poultry, such as chicks and ducklings, sourced from multiple hatcheries [5].

In 2011, a total of 68 individuals infected with the outbreak strain of *Salmonella* Altona were reported from 20 states [6]. Forty-two (74%) of 57 ill persons interviewed were reported to contact with live poultry (chicks, chickens, ducklings, ducks, geese, and turkeys) before becoming ill. Of ill persons who could recall the type of live poultry with which they had contact, 40 identified chicks, ducklings, or both, and 33 (89%) of 37 ill persons with available vendor information reported purchasing chicks and ducklings from multiple locations of a nationwide agriculture feed store. Traceback investigations of live chicks and ducklings from homes of ill persons identified the same single mail-order hatchery in Ohio identified in the outbreak of *Salmonella* Altona infections as the source of these chicks and ducklings. In June 2011, the Ohio Department of Agriculture inspected the mail-order hatchery and made recommendations for improvement [6].

Human infections with *Salmonella* sp. is a typical example of a recurring public health issue involving human illness linked to contact with asymptomatic animals (chicks, ducklings, chickens, ducks, turkeys, and geese) [7]. These illnesses are especially severe among young children who account for the majority of infections. Chicks and ducklings appear healthy and clean, but their bodies and areas where they live and roam can be contaminated with *Salmonella* sp., leading to human illness [3].

Microbial pathogens in animal feces can contaminate the environment in which animals are raised, where they roam, and where they are kept while awaiting slaughter [3]. Because animal hides and intestinal contents may have pathogens, efforts at slaughter are focused on cleaning the hides, removing them with care, and preventing the contamination of meat with intestinal contents. Poultry farms with large populations of birds are a setting where infectious agents can spread rapidly. When birds are slaughtered, hot water dips help remove feathers but can spread intestinal contents to subsequent carcasses [3].

The main contamination point throughout the meat production is the inadequate hygienic conditions and handling in slaughterhouses. The conditions before slaughter, such as feeding and housing, including spreadable contaminations from skin and feces, contents of digestion system, and contaminated water are sources of *Staphylococcus* sp., *Escherichia* *coli* and *Bacillus cereus* [8]. Different processes in slaughterhouses like evisceration can contaminate carcasses and equipment with gut bacteria [9].

Interestingly, it has been reported that bacterial diversity of environment samples in sheep slaughter line was higher than cattle [10]. This difference was due to the increased transmission from animal to the environment and to the production line probably due to manual slaughtering of sheep. On the contrary, in the cattle slaughter line all the slaughtering processes were performed on a production line with vertical rail dressing and automatic hide pullers and hygienic condition of bleeding in cattle slaughter line was better when the animals hoisted by one leg and bleeding continues until the blood flow was negligible. In general, contamination of carcasses was reduced by using automatic hide removal because there is less handling of the carcass and less use of knives [10].

After a large multistate *E. coli* O157:H7 outbreak was linked to undercooked ground beef patties sold from a fast-food restaurant chain, in 1993, *E. coli* O157:H7 became broadly recognized as an important human pathogen [11]. In 1994, officials at the USDA declared *E. coli* O157:H7 an adulterant of ground beef, so that finding these bacteria in ground beef resulted in its mandatory recall, and then implemented a new inspection procedure for beef carcasses based on Hazard Analysis Critical Control Point (HACCP) strategies [3]. In 2002, after a large multistate outbreak and recall of ground beef, regulators and slaughter and beef grinding companies focused more intensive effort on preventing the contamination of ground beef itself, including increased focus on hide removal, testing beef trim before it reached the grinder, and holding ground beef lots until they were found not to be contaminated. These efforts helped to reduce the contamination of ground beef and in turn may have led to the decrease in laboratory-confirmed *E. coli* O157:H7 cases measured in the US FoodNet active surveillance system [12]. Reducing these infections further will depend on pre-harvest interventions to decrease the shedding of *E. coli* O157:H7 by cattle before they come to slaughter. *E. coli* O157:H7 is common among cattle, particularly in the summertime, and reducing carriage may be achieved using a suite of interventions, including vaccines (two are currently available for evaluation), probiotics, and bacteriophage treatments, and microbicidal agents such as sodium chlorate [13].

In order to provide guidance to egg producers on certain provisions, FDA published in 2011 a document entitled “Prevention of *Salmonella Enteritidis* in Shell Eggs During Production, Storage, and Transportation” with guidance on how to implement *Salmonella Enteritidis* prevention measures, how to sample for *S. Enteritidis*, and how to maintain records documenting compliance with the final rule [14]. FDA's egg rule defines biosecurity as “a program, including the limiting of visitors on the farm and in poultry houses, maintaining personnel and equipment practices that will protect against cross contamination from one poultry house to another, preventing stray poultry, wild birds, cats, and other animals from entering poultry houses, and not allowing employees to keep birds at home, to ensure that there is no introduction or transfer of *S. Enteritidis* onto a farm or among poultry houses.” A similar program was launched in the United Kingdom in 1998 to reduce *Salmonella* infections. In the “British Lion” program, egg producers implemented measures voluntarily; including on-farm biosecurity, cleaning and disinfecting henhouses between flocks, vaccinating hens against *S. Enteritidis*, and monitoring them for the presence of infection [15].

Interestingly, in New Zealand, control measures implemented at slaughter led to a 50% reduction in campylobacteriosis in 2008 [16],[17]. In Scandinavia, a new control strategy is “test and freeze,” developed first in Iceland and then adopted in Norway and Denmark, in which flocks are tested pre-slaughter for the presence of *Campylobacter* sp. [18].

These egg safety programs typically included obtaining *Salmonella Enteritidis*-free chicks from hatcheries, preventing spread among flocks by biosecurity, cleaning and disinfection, and testing henhouse environments with diversion of eggs to pasteurization if *Salmonella Enteritidis* was found; these programs were associated with significant decreases in *Salmonella* infections [19].

Contamination of carcasses at slaughter has been found to be correlated to the prevalence of *E. coli* O157:H7 in cattle feces [20]. Many associations have been made between dietary factors and *E. coli* O157:H7 prevalence in cattle feces. Pre-harvest interventions, such as diet management, could reduce the fecal prevalence and diminish the impact of this adulterant. Dietary influences, including grain type and processing method, forage quality, and distillers grains have all been associated with *E. coli* O157:H7 prevalence. In addition, several plant compounds, including phenolic acids and essential oils, have been proposed as in-feed intervention strategies. The specific mechanisms responsible for increased or decreased *E. coli* O157:H7 shedding or survival are not known but are often attributed to changes in hindgut ecology induced by diet types. Some interventions may have a direct bacterial effect. Frequently, results of studies are conflicting or not repeatable, which speaks to the complexity of the hindgut ecosystem, variation in animal feed utilization, and variation within feed products. Understanding specific mechanisms, driven by diet influences, responsible for *E. coli* O157:H7 shedding will aid in the development and implementation of better and practical pre-harvest intervention strategies [20].

The CDC suggested that any contact with live poultry can be a source of human *Salmonella* infections, and published certain recommendations [6]. These included: (a) to wash the hands thoroughly with soap and water right after touching live poultry or anything in the area where they live and roam; (b) to clean any equipment or materials associated with raising or caring for live poultry outside the house, such as cages or feed or water containers; (c) to prevent children younger than 5 years of age, elderly persons, or people with weak immune systems from handling or touching chicks, ducklings, or other live poultry; (d) to prevent live poultry inside the house, in bathrooms, or especially in areas where food or drink is prepared, served, or stored, such as kitchens, or outdoor patios; (e) to prevent snuggle or kiss the birds, touch your mouth, or eat or drink around live poultry, and, for mail-order hatcheries, agricultural feed stores, and others who sell or display chicks, ducklings and other live poultry should provide health-related information to owners and potential purchasers of these birds prior to the point of purchase and these should implement interventions to prevent human *Salmonella* infections associated with contact with live poultry. Interestingly, 62% of case-patients reported contact with baby chicks or ducklings, and 45% were less than 10 years of age, and this finding is possibly attributable to the fact that children's immune systems are not fully developed and that young children typically have poor hand hygiene practices [21].

Apart from bacteria, viruses and protozoa may contaminate food, and a number of significant outbreaks have been attributed to theses parasitic microorganisms [2]. Among emerging parasitic infections that may be acquired by food are *Cyclospora cayetanensis*, *Giardia* sp., *Cryptosporidium* sp., *Fasciola* sp. and *Fasciolopsis* sp., while among the major foodborne parasites are *Toxoplasma gondii*, *Sarcocystis* sp., *Taenia* sp. and *Trichinella* sp. [22].

In 1993, Milwaukee, WI area experienced the largest documented water-borne disease outbreak caused by protozoa [23]. An epidemiologic investigation began after the health department was notified of gastrointestinal illness causing high absenteeism of hospital employees, students, and teachers. Within 4 days, oocysts were identified in residents' stools, treated water from one of the two water treatment plants was found highly turbid, a boil water advisory was issued, and that plant was closed. Oocysts were identified in ice made before and during the outbreak. Oocysts from Lake Michigan water apparently entered the southern treatment plant. Possibly, inadequate amounts of polyaluminium chloride or alum coagulant failed to reduce the high turbidity, and recycling of filter backwash water may have increased the number of oocysts in the finished water. Heavy rains, cattle manure on fields in the watershed, abattoir waste, and sewage overflow were considered potential sources [23],[24].

In addition, two outbreaks of toxoplasmosis, associated with the consumption of oocyst-contaminated water, have also been documented [25],[26]. The first one occurred in Panama, and epidemiological evidence indicated that the most likely vehicle for transmission was the ingestion of creek water, contaminated with oocysts excreted by jungle cats [25]. The second outbreak occurred in Canada, 110 acute *Toxoplasma* sp. infections were identified, and the epidemiological evidence showed that a waterborne source was implicated, whose water was probably contaminated with oocysts from domestic and feral cats and cougars [26].

Activities associated with cattle farming, e.g. slurry spraying and run off from contaminated grazing land, have been proposed as causes of many of parasite-caused outbreaks, but, in the absence of definitive information in many instances, the number attributed to the zoonotic route has to remain speculative [27],[28].

Parasites may be transmitted by fish, reptiles, amphibian, snails, crustaceans and bivalves [22]. Bivalves act as transport hosts by concentrating viable *Cryptosporidium* sp. oocysts and *Giardia* sp. cysts (and probably other zoonotic transmissive stages found in fecally-contaminated fresh, estuarine and marine waters) from their environment and have been suggested as reservoirs for zoonotic transmission [27]. Infection can be clinical in calves, but subclinical in adult cattle. A clinically ill neonate can excrete approximately 10^9^ oocysts daily during the course of infection, whereas a clinically-well, infected cow can excrete between 7.6 × 10^5^ and 7.2 × 10^8^ oocysts daily [27].

Traditionally, these parasitic zoonoses are most common in Asia because of the particular food practices and the importance of aquaculture [22],[29]. However, some of these parasites may emerge in other continents through aquaculture and improved transportation and distribution systems to bring aquatic foods to local and international markets, changing culinary practices and increased tourism [22],[29].

Interestingly, molluscan shellfish filter large quantities of water, extract tiny particles that remain on their gills and thereby make excellent biological indicators of water-borne pathogens [2]. Oocysts of *C. parvum* have been detected in oysters, clams, and mussels collected from the Chesapeake Bay [30],[31], in mussels from the coast of Ireland [32], and in oysters from Galicia, Spain [33]. Although none of these findings were associated with outbreaks of cryptosporidiosis, repeated outbreaks of viral and bacterial illness associated with ingestion of raw shellfish should serve as a warning that cooking of shellfish will reduce the risk of illness from all these pathogens.

In Thailand, a FAO led HAACP approach to fish pond management was carried out that focused on water supply, fish fry, fish feed and pond conditions to eliminate contamination of the ponds with *Opisthorchis* *viverrini* eggs and snail infections. A preliminary report indicated some success with this intensive effort, but a full assessment of its sustainability over a period of years is needed [34],[35]. A code of practice for fish and fisheries products has published by FAO/WHO aiming to provide a user-friendly document as background information and guidance for the elaboration of fish and shellfish process management systems that would incorporate good manufacturing practice (GMP) as well as the application of HACCP in countries where these, as yet, have not been developed. In addition, it could be used in the training of fishers and employees in the fish and shellfish processing industries [36].

Animal manure is a recognized source of anthropozoonotic parasites such as *Cryptosporidium* sp. and is also a favorite breeding place, food source, and landing site of filth flies [37]. *Cryptosporidium parvum* oocysts can be transported by filth flies not only from cattle sources but from any unhygienic or contaminated source, i.e., toilets, abattoirs, trash, carcasses, and sewage [37]. Because wild filth flies carry viable *C. parvum* oocysts acquired naturally from unhygienic sources, they can be involved in the epidemiology of cryptosporidiosis [37]. Filth flies can cause human or animal cryptosporidiosis via deposition of infectious oocysts on visited foodstuff; however, such epidemiologic involvement is difficult to prove [37].

Viruses are particulate in nature and multiply only in other living cells. Thus, they are incapable of survival for long periods outside the host. More than 100 types of enteric viruses have been shown to cause foodborne illness; the most common foodborne virus pathogens are Hepatitis A and Noroviruses [2]. These viruses are frequently transmitted via food; bivalve molluscs, such as clams, cockles, mussels, and oysters, are especially prone to transmit viruses. The waters in which they grow are increasingly subject to human fecal contamination, sometimes from sewage discharges and sometimes from infected shellfish harvesters. The shellfish collect viruses in the course of their filter feeding activity. Human viruses do not infect these species, but they are harbored for days or weeks in the shellfish digestive tract and are apparently more difficult to remove than bacteria during processes intended to cleanse the shellfish (e.g. depuration) [2].

Bivalve molluscan shellfish are notorious as a source of foodborne viral infections, because filter-feeding shellfish can concentrate hepatitis A up to 100-fold from large volumes of water, allowing accumulation of virus from fecally contaminated water [38]–[40]. Lees has reviewed the association of viruses with bivalve shellfish [41]. Interestingly, contamination may occur at almost every step in the path from farm to table. Outbreaks associated with food, particularly fresh produce, contaminated before reaching the food service establishment have been recognized increasingly in recent years [42],[43]. This produce appears to have been contaminated during harvest, which could occur from handling by virus-infected individuals.

## 3. Outbreak investigations—non-animal products

Large outbreaks of human infections linked to fresh produce consumed after minimal processing have been more frequently identified in recent decades [44],[45]. There is little that consumers can do to protect themselves because these foods are not cooked, washing them has little effect on contamination, and may contaminate other foods during food preparation, especially in salads and sandwiches. Therefore, it is particularly important to prevent such contamination from happening in the first place.

In July 1995, 40 Montana residents were identified with laboratory-confirmed *E. coli* O157:H7 infection and 52 residents had bloody diarrhea without laboratory confirmation [46]. A case-control study showed that 70% of patients and only 17% of controls reported eating purchased (not home-grown) leaf lettuce before illness. The environmental investigation included the implicated local produce farm and area grocery stores, examining leaf lettuce growing, harvesting, and handling practices at the farm; delivery and distribution practices from farm retail market; and leaf lettuce handling procedures within retail stores [46]. Although it was not known how exactly contamination of leaf lettuce occurred, it has been suggested at least four possibilities [46]: (A) The farm fertilized its leaf lettuce with compost that contained manure obtained from a local dairy; studies of cattle herds have shown that approximately 0.3% of cattle carry *E. coli* O157:H7 [47]; if compost was contaminated with *E. coli* O157:H7 and gained access to the fields, it could have directly contaminated the produce, as has occurred in the past [48]. (B) If infected cattle feces were present in the adjacent uphill pasture, these feces could contaminate either the water used for irrigating the fields (flood irrigation) or surface water runoff, which could then contaminate the lettuce. (C) Since cattle had access to the streams above the pond used for irrigating the lettuce, their feces could have contaminated this water directly. (D) Feces of other animal reservoirs of *E. coli* O157:H7, such as the sheep kept on the farm or deer, could also have contaminated irrigation water or the lettuce [49],[50].

In 2006, a multi-state outbreak of approximately 200 illnesses with *E. coli* O157:H7 infection from 26 states was linked to the consumption of fresh spinach [51]. An environmental investigation identified *E. coli* O157:H7 isolates with a pulsed-field gel electrophoresis (PFGE) pattern indistinguishable from the outbreak strain in samples obtained from river water, cattle manure, and wild pig feces in and around a field used to grow one brand of spinach from the implicated lot [52].

An instructive outbreak of produce-related illness linked to wildlife intrusion was identified in Alaska in 2008 [53]. Raw peas had been suspected as the source of a small cluster in 2005, and a larger increase in 2008 was rapidly shown to be associated with eating raw peas, from one local farm, which was adjacent to a nature preserve for the Sandhill Crane, *Grus canadensis.* Peas were harvested mechanically and washed in a tank without added chlorine. After harvest, shelled peas were bagged and labeled with directions for blanching, though they were often repacked in bags without this advice, and eaten without blanching. Cranes were observed feeding on peas in the growing fields at the time of harvest, and molecular subtyping studies confirmed that some *Campylobacter* bacteria isolated from patients were indistinguishable from strains isolated from peas, and from crane feces. This investigation shows that wild birds may be an under recognized source of produce contamination, and that some basic prevention measures may make it safer. Animal intrusions have also been suspected as the likely source of contamination of apples in cider orchards by cattle or deer with *E. coli* O157:H7 and *Cryptosporidium* sp. [54],[55] strawberries by deer with *E. coli* O157:H7 [56], where the investigation identified fresh strawberries as a novel vehicle for *E. coli* O157:H7 infection, implicated deer feces as the source of contamination, and highlights problems concerning produce contamination by wildlife and regulatory exemptions for locally grown produce. An outbreak of verotoxin-producing *E. coli* in Sweden caused by the consumption of lettuce that was irrigated by water from a small stream was investigated [57]. Identical verotoxin-producing *E. coli* O157:H7 strains were isolated from the patients and in cattle at a farm upstream from the irrigation point. An *E. coli* O157:H7 outbreak in the US associated with shredded lettuce was traced back to the accidental mixing of well water, intended for irrigation, with water from a dairy manure lagoon [58].

Consumption of vegetables from a manured garden caused an *E. coli* O157:H7 infection in Maine and the same strain of *E. coli* O157:H7 was cultured from both the patient and manure from the garden [48]. In 1991, an outbreak of *E. coli* O157:H7 infections associated with consumption of unpasteurized apple cider was attributed to use of apples collected from the ground that may have become contaminated by manure [54].

Another case where irrigation water was implicated in outbreaks of *E. coli* O157:H7 infection from contaminated lettuce was reported [46]. The farm obtained its irrigation water from a nearby pond supplied by several streams that passed through cattle fields. Sampling of water and feces did not yield *E. coli* O157:H7. However, the environmental sources of potential water contamination were present, including improperly aged compost, feces of possibly infected cattle in the adjacent uphill pasture, cattle access to the streams above the pond used for irrigating the lettuce, and feces of other animal reservoirs of *E. coli* O157:H7, such as the sheep kept on the farm or deer. Irrigation water was also implicated in an outbreak of *E. coli* O157:H7 attributed to mesclun lettuce, which was suspected to have been irrigated with water contaminated by dust from cattle grazing land [59]. At the time of the environmental investigation, however, no *E. coli* O157:H7 was isolated from samples of well water, water from a cattle trough, water sampled from the cattle pasture, and cow or chicken manure. Irrigation water was also the source that may have contributed to contamination of the spinach and hence to the multistate E. coli O157:H7 outbreak associated with spinach in 2006 and traced to California [60].

Irrigation water was also implicated as a source of *E. coli* detected on cabbage seedlings irrigated with water inadvertently contaminated by a municipal sewage release; no *E. coli* were detected on seedlings in an adjacent field irrigated with municipal water [61]. Although the source of the crop contamination could not be demonstrated conclusively because water samples tested negative for *E. coli*, the authors speculated that the creek water used for irrigation contained pathogenic bacteria associated with human waste or waste from wild animals [61].

Parasites can contaminate food at any stage of the food chain, particularly vegetables and fruits, are rinsed in parasite-contaminated potable water at some point throughout the food chain. Surface contamination can be direct, following contamination by the infected host, or indirect, following contamination by transport (birds, flies, etc.) hosts, the use of manure and contaminated water for irrigation, fumigation and pesticide application, etc [49]. The risk of foodborne transmission has increased nowadays, since consumers' habits have shifted to the consumption of raw vegetables and undercooking to retain the natural taste and preserve heat-labile nutrients [49].

Foodborne outbreaks caused by viruses and linked to fresh produce consumption was reviewed and reported that one hundred and fifty two viral outbreaks linked to fresh produce consumption were identified [62]. The majority of the reported outbreaks was reported in Europe, followed by North America, Asia, Australia, Africa and South America. The most common viral pathogens were norovirus (48.7%) and hepatitis A virus (46.1%) and the most frequent type of fresh produce involved was frozen raspberries (23.7%) [63].

A viral outbreak was reported and attributed to the consumption of apple cider [64]. The fresh pressed cider was squeezed from apples collected from an orchard in which an infected calf grazed. Some apples had fallen onto the ground and had probably been contaminated with infectious oocysts [64]. In Maine, US, apples from the ground near a cattle pasture were used for cider at an agricultural fair and 160 attendees developed cryptosporidiosis [65]. Oocysts have been found on the surface of raw vegetables from the market place. Cool, moist vegetables provide an optimal environment for survival. In Costa Rica oocysts were found on cilantro leaves and roots, lettuce, radishes, tomatoes, cucumbers, and carrots but not cabbage [23]. In a suburban slum of Lima, Peru, basil, cabbage, celery, cilantro, green onions, ground green chilli, leeks, lettuce, parsley, and yerba buena from several markets were contaminated with oocysts of *C. parvum* [23]. Vegetables can be contaminated from fertiliser of animal or human feces; by contaminated water used to irrigate or moisten produce; by soiled hands of farm workers, produce handlers, or food workers; and from contaminated surfaces where vegetables are packed, stored, sold or prepared. It should be noted that, in most protozoan outbreaks, the absence of standardised detection and subtyping methods limits our understanding of the zoonotic route of infection [27].

Parasites can contaminate crops through various routes, for example, via water contaminated feces that is used for irrigation or spraying of crops, by poor personal hygiene practices among pickers or handlers of crops, by contact with contaminated soil or by contact with feces of wild animals. The relative importance of these routes is unknown, although contamination by wild animals is not likely for *Cyclospora* sp.. Even with the well-studied outbreaks of cyclosporiasis that have been traced to Guatemalan raspberries, the exact route of the contamination remains a matter of speculation, although irrigation water or insecticides and fungicides made with contaminated water used to spray crops seems to be a possible cause [66],[67].

Foods become contaminated either directly by infected people or through sewage pollution. Those enteric viruses, which are commonly associated with foodborne outbreaks, either cannot be cultured in the laboratory or can only be cultured with difficulty. Hence information and experimental studies on survival and recovery of viruses from foods often relates to other virus types that are readily cultured. They infect via the gastrointestinal tract, occur in the environment as a result of sewage contamination and are relatively stable [68]. Fruits and vegetables may become contaminated with viruses in two ways. First, they may be contaminated in their growing area before harvest by coming into contact with inadequately treated sewage or sewage polluted water. Secondly, contamination can arise during processing, storage, distribution or final preparation either directly from infected people or by contact with a contaminated environment [68]. In most outbreaks of foodborne viral disease involving fresh produce, it is not known whether contamination took place before, during or after harvest [68]. The transmission of viruses is thought to be mainly by surface contamination. There are relatively few reported studies on the possible uptake of viruses within damaged plant tissues during primary growth. Studies with poliovirus report that virus can infiltrate into the roots and body of plants from the soil [69], but there is no evidence of illness from this source. Viruses from sewage do not bind readily with soil particles and can enter groundwaters leading to contamination of water sources. The viruses causing gastroenteritis and hepatitis A appear to be extremely infectious in very low doses.

Human enteric viruses can potentially be present in any type of water contaminated by human fecal material and by sewage. Mounting evidence suggests that viruses can survive long enough and in high enough numbers to cause human diseases through direct contact with polluted water or contaminated foods [70],[71]. When hepatitis A virus was detected in lettuce from Costa Rica, it was suggested that the possible source of contamination was the discharge of untreated sewage into river water used to irrigate crops, which is common practice in some less well-developed countries [72]. Certainly, climate, the nature of the soil and the nature of the resident microflora determine virus survival and retention within soil particles [73].

An outbreak involving contaminated lettuces by wild animals with *Yersinia pseudotuberculosis* was investigated in Finland [74]. A total of 287 samples were collected from lettuce, surface soil, animal feces found in the fields, and the irrigation water system (51 from soil, 21 from sludge, 22 from feces, 39 from water, 128 from lettuce, 4 from compost, and 22 from water pipes). No implicated iceberg lettuce was available for culture by the time the trace-back investigation had been completed due to weather conditions. No animal manure was used as fertilizer, and there were no domestic livestock near the fields. Untreated water was used for spray irrigation of the fields. Fields were unfenced, and wildlife had free access to irrigation water sources and fields. No evidence of the presence of unusual numbers of small rodents or lagomorphs on the farms was found. However, an ecologically distinct feature of the southwest archipelago is a large population (110,000 animals) of nonnative roe deer (*Capreolus capreolus*) that were introduced to the islands during the 1960s. Large quantities of roe deer feces were found all over the lettuce fields and around all irrigation water sources. Of the 287 samples obtained, 72 (25%) yielded bacteria of *Yersinia* sp. *Y. pseudotuberculosis* was recovered from one soil and from one irrigation water sample. The exact mechanism for contamination of the iceberg lettuce remains unknown, but it is likely to have resulted from use of irrigation water contaminated with animal feces [74]. It could also have occurred from direct contamination by animal feces or from surface water runoff. The traceback investigation suggested that contamination was probably intermittent and not uniform. Wildlife had access to irrigation water sources and fields, and large quantities of roe deer feces were found in both areas. In Europe, Japan, and North America, *Y. pseudotuberculosis* is frequently isolated from many domestic and wild animals [75]. Although the recovered environmental isolates were not related to the outbreak, the presence of *Y. pseudotuberculosis* bacteria in iceberg lettuce and irrigation water samples indicated fecal contamination from infected animals, supporting the hypothesis that contamination occurred at the farm before distribution [74].

Water contaminated with animal feces and then used to irrigate plants has also been a route connecting plant production with animal reservoirs. In 2006, an outbreak of approximately 80 persons with *E. coli* O157:H7 infection was linked to lettuce served at locations of a Mexican-style fast food restaurant chain in Iowa and Minnesota [76]. An investigation identified dairy farms near lettuce fields in California that provided lettuce to the restaurants where ill persons had eaten. The 31% of environmental samples tested were positive for *E. coli* O157:H7 and determined to genetically match the restaurant outbreak strain [76]. As for the source tracking, the irrigation system was connected to the dairy wastewater blending and distribution system, with inadequate backflow protection devices, presenting a possible route for contaminated water to be used on fields adjacent to the lettuce-growing fields associated with this outbreak. These findings indicated that the nearby dairy farm was the likely source of this outbreak [76].

A total of 72 culture-confirmed *S. Newport* isolates with indistinguishable PFGE patterns were identified from stool specimens collected during July to November 2005 in 16 states in the US. Illness was associated with eating raw, large, red, round tomatoes at restaurants. Implicated tomatoes had been purchased whole and sliced at restaurants. No single restaurant or restaurant chain was associated with the outbreak [77]. Investigators determined that the implicated tomatoes were grown on two farms on the eastern shore of Virginia. The outbreak strain of *S. Newport* was isolated from irrigation pond water near tomato fields in this region in October 2005 [77].

Cantaloupe-associated outbreaks that were reported to the Centers for Disease Control and Prevention (CDC) were reviewed [78]. Twenty-three outbreaks between 1984 and 2002, where 1434 people became ill, 42 were hospitalized, and two died in these outbreaks. It was concluded that soil and soil amendments such as improperly composted manure, contaminated irrigation water, wild and domestic animals, and farm workers are potential vehicles of contamination of pre-harvest melons, and that microorganisms capable of causing human diseases can survive in soil for protracted durations [79]. *Listeria monocytogenes* can survive in soil for at least 8 weeks, *Salmonella* sp. and *E. coli* O157:H7 can survive up to 23 weeks, and viruses can live for 3 weeks. These pathogens may also be introduced by infected or colonized wild animals, such as reptiles, birds, and rodents, eating fruit and defecating directly in fields, and further distributed by insects and perhaps nematodes [78].

Sprout-associated outbreaks represent a special scenario, in which the presence of even a few bacterial cells on seeds can be amplified to a large number as a result of the sprouting process itself [79]. As seeds are a raw agricultural commodity rather than a processed food, they may not be expected to be free of pathogens, and their transformation into a food (the sprouts themselves) actually increases the risk, unless special measures are taken to decontaminate the seeds before sprouting and to regularly test the sprouting environment for contamination. Sprouts have been identified as a special food safety problem because of the potential for pathogen growth during the sprouting process. If pathogens are present on or in the seed, sprouting conditions may favor their proliferation. There is no inherent step in the production of raw sprouts to reduce or eliminate pathogens. Contaminated seed is the likely source for most reported sprout-associated outbreaks [80].

In 2011, an outbreak caused by a rare strain of *E. coli* O104:H4 was reported in Germany [2],[81]. This was the second largest and the deadliest outbreak of *E. coli-*associated disease ever recorded. Between May 21 and July 22, 2011, more than 4,000 people became ill in 16 countries, and 50 individuals died [2]. By the time the outbreak ended in early July, 2011, there were reports of more than 4,000 illnesses, 800 cases of hemolytic uremic syndrome (HUS), and 50 deaths in Germany and 15 other countries [2],[82]. Interestingly, investigators initially identified fresh produce—including leafy greens, tomatoes, and cucumbers as likely sources [83]. Traceback studies of disease clusters in five German provinces that were affected early in the outbreak pointed to sprouts produced by an organic grower in Germany [2],[84].

## 4. Survival of pathogens on the plant tissues

Laboratory and field studies showed that foodborne pathogens transmitted from irrigation water to fresh produce can remain viable for variable periods of time, depending on environmental conditions [85]. Interestingly, *E. coli* persisted for up to 28 days whereas *E. coli* O157:H7 did not survive for more than 14 days in inoculated spinach plants [86]. Survival of foodborne pathogens is augmented by inclusion in plant phyllosphere biofilms or internalization within the plant [87].

Similar to plant-associated bacteria, pathogenic bacteria use cellulose and aggregative fimbriae for their attachment to plant surfaces [88],[89]. It has been observed that the transfer of *Salmonella* sp. to parsley leaves via irrigation water was dependent on curli forming abilities of the strains [89]. Similarly, significantly higher attachment of curli-expressing *E. coli* O157:H7 on iceberg lettuce and cabbage than the attachment of curli-negative *E. coli* O157:H7 strains has been reported [90]. Any pathogen may reach plant surfaces via irrigation water; however the potential for adherence is both strain and plant specific. For example, strain-specific properties of *Salmonella* sp. (curli and cellulose) affected its ability to enter parsley plants from contaminated irrigation water [89].

Recent research indicates that fresh produce can serve as an important vehicle for transmission of foodborne pathogens [91]. However, better understanding on interactions between foodborne pathogens and plants or vegetables has become increasingly important. In particular, factors influencing fitness of human pathogens including survival/colonization in plants and molecular mechanisms of plant defense responses need to be elucidated [92].

Recently, *Arabidopsis thaliana* challenged with *E. coli* O157:H7 and *Salmonella* *Typhimurium* was shown to induce salicylic acid-dependent plant defense responses [93],[94]. Bacterial cell surface structures of *E. coli* O157:H7 and *S*. *Typhimurium*, including ﬂagella, curli, and extracellular polysaccharides, have been shown to have a role in activation of plant defense responses, thereby restricting bacterial colonization on plants [95]–[97]. However, influence of bacterial surface structures and plant defense responses on the plant-pathogen interactions remains largely unknown. Several studies have reported physiology, virulence factors, pathogenicity of *E. coli* O104:H4 [98]. Understanding survival mechanism of *E. coli* O104:H4 on plant tissue is important in designing control strategies for fresh produce safety. However, very little is known about behavior of *E. coli* O104:H4 on plant systems, particularly with respect to plant defense response. Recently, the survival of *E. coli* O104:H4 strains compared with *E. coli* O157:H7 strains on *Arabidopsis thaliana* was investigated, as well as, on romaine lettuce [99]. They determined induction of plant defense response that regulates microbial survival/persistence to understand molecular responses of plants to STEC strains. The populations of *E. coli* O104:H4 and *E. coli* O157:H7 strains on both *Arabidopsis thaliana* and lettuce were shown to gradually decline over 5 days, which indicates that the STEC strains might have less fitness to plant. In terms of comparison in survival ability between *E. coli* O104:H4 and *E. coli* O157:H7, *E. coli* O104:H4 strains survived better compared with *E. coli* O157:H7 strains on both *Arabidopsis thaliana* and lettuce at day 5 post-inoculation. Similarly, it has been reported that *E. coli* O104:H4 was detectable at 10-day on basil plant whereas *E. coli* O157:H7 did not recover, indicating *E. coli* O104:H4 isolates may have enhanced fitness to plant [100].

## 5. Conclusions

**Figure 1.**
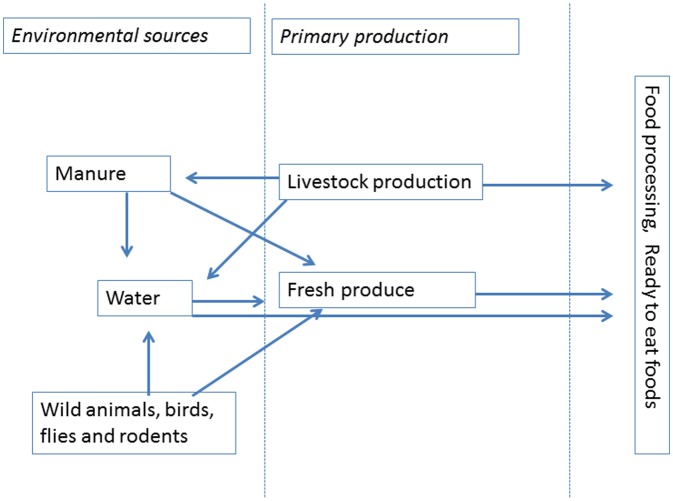
Α schematic presentation of the possible routes of contamination from polluted sources at the early stages of the food chain.

Outbreak investigations have revealed direct links between fresh produce, animal reservoirs and water used for irrigation and for raising animals in livestock. Several produce associated outbreaks have followed wildlife intrusion into growing fields or fecal contamination from nearly animal production facilities that likely led to produce contamination. The sources of contamination with microorganisms at the early stages of fresh produce and meat production are, basically: (1) water, used for irrigation and application of pesticides; (2) manure, applied as fertilizer; (3) livestock, wild animals, birds, flies and rodents. The possible routes for contamination are shown in Figure 1.

Food safety depends on understanding these pathways well enough to prevent them at the early stages of the, anyway complicated, food chain. In order to minimize the risk for microbial contamination from polluted sources, modern agricultural systems, similarly to modern processing systems should follow guidelines for GMP and good agricultural practices (GAP). A great number of guidelines for fresh produce have been published [101]–[108]. It can be concluded that stakeholders, that is governments and industry should develop education programs for fresh produce and raising animal producers on basic principles for microbiological food safety. GAP, GMP and HACCP systems should be implemented to reduce the potential for microbial contamination during the early stages of the food chain.

Risk assessment, in general, is the characterization and estimation of potential adverse health effects associated with exposure of individuals or populations to hazardous materials or situations. With regard to irrigated produce, adverse health effects may be caused by the ingestion of pathogens with the produce, by inhaling aerosols containing pathogens, by the unintended consumption of contaminated water, etc. Quantitative microbial risk assessment (QMRA) has been first applied to wastewater irrigation and currently is actively applied to irrigation with water from other sources [109]. The QMRA establishes a relationship between the concentrations of pathogenic microorganisms in irrigation water and the probability of illness. Comprehensive introductions in QMRA in general and in irrigation QMRA have been published [109],[110].

Preventive measures, as part of a GAP system, should include preventing animals from entering streams by fencing or using off-stream water sources in riparian areas, being aware of upstream use and sources of water that are planned to be used in irrigation, and preventing manure run-off from fields, pastures, and feedlots to irrigation water sources as a part of GAP [111]. Management practices have been recommended that limit the probability that feedlot cattle shed foodborne pathogens in their feces, dietary interventions affecting pathogen shedding in cattle [20],[112].

If vegetables are grown next to an animal-rearing operation, there is a potential for product to become contaminated, directly, or indirectly, by animals, run-off, bio-aerosols, or vectors associated with the animal operation such as birds, rodents, and flies [60],[113]. By use of clean or treated irrigation water and by protecting the fields and water sources from animals, preventing animals from entering streams by fencing could minimize the risk from nation.

Much of the progress in risk factors of contamination with human pathogens has been focused on safer processing of animals and plants after they are harvested, with less emphasis on the prevention that can be achieved before harvest or slaughter, that is at the early stages of the food chain. Controlling and minimizing pre-harvest contamination may be one of the key aspects of food safety.
